# Comparative Bioavailability Study of a Novel Multi-Day Patch Formulation of Rivastigmine (Twice Weekly) with Exelon^®^ Transdermal Patch (Daily)- A Randomized Clinical Trial

**DOI:** 10.2174/1567205019666220823105059

**Published:** 2022-11-10

**Authors:** Bjoern Schurad, Cornelius Koch, Barbara Schug, Adelaida Morte, Anna Vaqué, Rafael De la Torre, Marc Iniesta

**Affiliations:** 1 Luye Pharma AG, Miesbach, Germany;; 2 SocraTec R&D GmbH, Erfurt, Germany;; 3 ESTEVE Pharmaceuticals SA, Barcelona, Spain;; 4 Research Group in Integrated Pharmacology and Systems Neuroscience, Hospital del Mar Research Institute Doctors (IMIM), Barcelona, Spain

**Keywords:** Alzheimer's disease, rivastigmine, transdermal patch, bioavailability, bioequivalence, healthy subjects, butyrylcholinesterase, pharmacokinetics

## Abstract

***Background*:** Rivastigmine, a reversible AChEI for symptomatic treatment of mild to moderately severe Alzheimer’s dementia, is administered once daily transdermal patches, enabling an easier and continuous drug delivery. A novel multi-day (twice week) patch formulation was developed with greater convenience for patients’ therapeutic management.

***Objective*:** To assess the bioequivalence under SS conditions of the multiple-day rivastigmine transdermal patch (Test Product, RID-TDS) in comparison to the once-daily Exelon^®^ transdermal patch (Reference Product), both at a release rate of 9.5 mg/24 h.

***Design*:** Single-center, open-label, randomized, multiple-dose study in healthy male adults in a 2-period, 2-sequence-crossover design with multiple applications.

***Methods*:** Patches were applied on 11 consecutive days for Exelon^®^ and a 4-3-4-day regimen for the multiday test patch (RID-TDS), separated by a 14-day wash-out period. The safety, local tolerability and inhibitory effect of rivastigmine on plasma BuChE activity were also evaluated.

***Results*:** 57 subjects completed the study according to the protocol. Calculated point estimates and 90% CI for all primary parameters (AUC_96-264_, Cmax_96-264_ and Cmin_96-264_) were within the predefined acceptance interval of 80.00-125.00%. They were 113.64% (107.33-120.33), 105.14% (98.38- 112.38) and 107.82% (97.78-118.89) respectively. Satisfactory adhesion (CI of mean adhesion above 90%) was demonstrated for RID-TDS but not for Exelon^®^.

***Conclusion*:** Bioequivalence was demonstrated between RID-TDS mg twice a week and Exelon^®^ once daily in SS. Patch adhesion favored RID-TDS despite the longer dosing interval. Both products were well tolerated.

***Trial Registration Number*:** Protocols are registered in ClinicalTrials.gov: NCT03659435 and EudraCT: 2018-001570-18.

## 1 INTRODUCTION

Alzheimer's disease (AD) is one of our century's greatest medical care challenges and is the main cause of dementia [1]. The basic pathophysiology and neuropathology of AD suggest that the primary histopathologic lesions are the extracellular amyloid plaques and the intracellular Tau neurofibrillary tangles (NFTs) that result in progressive neuronal destruction leading to shortage and imbalance between neurotransmitters [2]. The progressive loss of limbic and neocortical cholinergic innervation in AD is critically important for the decline of memory, learning, attention, and other higher brain functions.

All of the already established treatments that are used today try to counterbalance this neurotransmitter imbalance. Acetylcholinesterase inhibitors (AChEIs) increase the availability of acetylcholine at synapses and have been proven clinically useful in delaying the pathologic cognitive decline in AD [3].

Rivastigmine is a reversible AChEI available for symptomatic treatment of mild to moderately severe Alzheimer’s dementia management. Rivastigmine transdermal patches enable an easier and continuous drug delivery in the longterm management of AD. Patch formulations avoid gastrointestinal absorption and hepatic first-pass metabolism, reduce adverse effects related to peak plasma drug concentrations and facilitate patient compliance. This is especially relevant for elderly people, who are often polymedicated and have difficulty swallowing, in which transdermal drug delivery can be a good alternative route of administration [4]. In a recent pharmacoeconomic report, rivastigmine transdermal patch was shown to be the most cost-effective pharmacological treatment option in AD [5].

Currently, transdermal rivastigmine patches are commercially available for a once-daily application. Advancing in the development of novel formulations with greater convenience and ease for the therapeutic management of patients, Luye Pharma AG has developed a rivastigmine multi-day patch formulation. Rivastigmine multi-day patch employs an innovative drug delivery system for rivastigmine, designed to deliver the drug constantly over an enhanced period of up to 4 days, allowing twice-weekly transdermal administration. For the convenience of use, it is targeted that the patch is replaced alternating after 4 days / 3 days, enablingtwo fixed days of patch change a week, *i.e.* each Monday and Friday morning. The twice-a-week patch will require a less frequent application presenting a better guarantee of receiving rivastigmine constantly and facilitating the treatment compliance of the polymedicated patient. Additionally, it should be associated with a need for less planning and logistics of the patients and caregivers (mainly during the weekend, that is, avoiding the need for patch application during this interval) and thus less burden on the caregiver. Patient and caregiver satisfaction is essential for good adherence to treatment.

The present study was designed to demonstrate the bioequivalence at steady-state after multiple doses administration of the new twice-weekly patch (RID-TDS 9.5 mg/24 hours) and the marketed daily reference product Exelon^®^ transdermal patch (9.5 mg/24 hours) under steady-state conditions. The study objectives to evaluate the safety, local tolerability and inhibitory effect of rivastigmine on plasma butyrylcholinesterase (BuChE) activity.

## 2 MATERIALS AND METHODS

### 2.1 Study Design

This is a Phase 1, single-center, open-label, with randomized order of treatments, balanced, 2-period, 2-sequence, cross-over study with multiple applications of rivastigmine transdermal patches to evaluate and compare the bioavailability and therefore to assess the bioequivalence under steady-state conditions of two different formulations of rivastigmine. A confirmatory assessment of the Test Product was conducted according to EMA requirements of adequate adhesion properties.

The study was conducted at SocraTec R&D GmbH - Clinical Pharmacology Unit (Mainzerhofplatz 14 99084 Erfurt, Germany). The study was reviewed and approved by the Ethics Committee of the Landesärztekammer Thüringen (august 2018) and conducted in accordance with the ethical principles originating from the Declaration of Helsinki and amendments and the International Conference on Harmonization Good Clinical Practice guidelines and local regulatory requirements. All subjects provided signed informed consent. Protocols are registered in ClinicalTrials.gov: NCT03659435 and EudraCT: 2018-001570-18.

### 2.2 Inclusion and Exclusion Criteria

Following the recommendations of the Guideline on the Investigation of Bioequivalence (European Medicines Agency, EMA, 2010), healthy subjects of both sexes aged 18 years or older and of weight within the normal range according to accepted normal values for the BMI (*i.e.* ≥ 18.5 kg/m^2^ and ≤ 30.0 kg/m^2^) should be included. Only male subjects were included as women present higher susceptibility to gastrointestinal adverse reactions associated with rivastigmine although no gender effect was evidenced for the administration of rivastigmine *via* the transdermal route [6, 7]. A lower limit of at least 50 kg was determined for the body weight of participating subjects due to safety reasons. According to the available data, patients with body weight below 50 kg may experience more adverse reactions and, on these grounds, may be more likely to discontinue treatment. The age of participants was limited to a maximum of 55 years for reasons of safety, as it was expected that by this measure eventual occurrence of treatment-emergent urinary obstruction would be limited, as cholinomimetics may induce/exacerbate urinary obstruction in men [8]. Therefore, healthy male adults aged 18–55 years with body mass index ≥18.5 and <30.0 kg/m^2^ were eligible for the study if they were non- or ex-smokers and in good health as determined by medical history review, physical examination, vital signs, electrocardiogram (ECG) and clinical laboratory tests. The exclusion criteria were chosen to avoid safety concerns related to the administration of rivastigmine and to minimize any impact on pharmacokinetic parameters. Additionally, subjects with skin conditions or abnormalities that could affect dermal absorption were considered non-suitable to be included in the study.

### 2.3 Study Treatments

The study treatments were: RID-TDS 9.5 mg/24 h by Luye Pharma AG (Test Product) and Exelon^®^ 9.5 mg/24 h by Novartis Pharma (Reference Product). The Test Product, offering a nominal content of 51.84 mg rivastigmine and an area of 21.6 cm^2^, was developed to deliver 9.5 mg/24h. An adhesive cover is applied over the patch to secure attachment to the skin. The Reference product offers a nominal content of 18 mg, an area of 10 cm^2^ and a labelled release rate of 9.5 mg/24h.

**Fig. (1):**
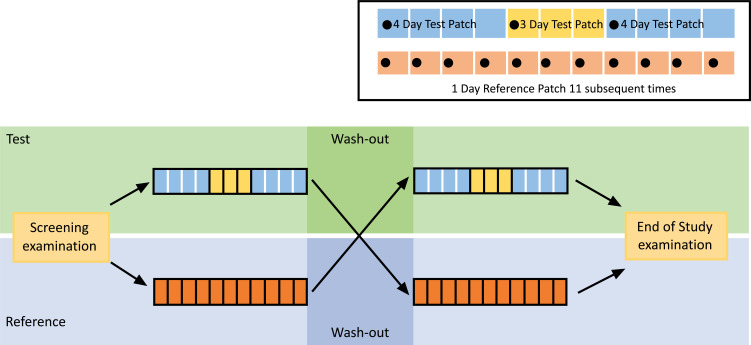
Study design diagram (Test = Treatment A; Reference = Treatment B).

Transdermal patches were consecutively applied in this clinical trial within each treatment (Fig. **1**). In the case of the Test product, 3 transdermal patches with a nominal release rate of 9.5 mg/24 h each (first patch to be applied for 4 days, second patch to be applied for 3 days and third patch to be applied for 4 days) were consecutively applied over a period of 11 days. In the case of the Reference product, 11 transdermal patches with a nominal release rate of 9.5 mg/24 h each were consecutively applied over a period of 11 days. Thus, the resulting total dose applied per treatment was 104.5 mg rivastigmine.

Patches were applied to clean, dry, hairless, intact, healthy skin of the upper back. The investigational patch application was standardized for all subjects, and the site was different within the same study period since no overlapping of the same application site should occur within a minimum of 14 days. Immediately after the test product was applied, the adhesive cover was placed over it to secure the proper attachment to the skin. After the application of the Reference product, no special adhesive cover was required.

A washout period of at least 14 days was observed between the last patch removal of the first treatment (study day 12 in the period I) and the first patch application of the subsequent treatment (study day 1 period II).

Blood samples (Table **1**) were collected in 6mL vacuum collection heparin tubes from a vein using an indwelling cannula with a switch valve or by direct venipuncture.

The compound rivastigmine was quantified using a validated analytical method using liquid chromatography coupled to mass spectrometry (LC-MS/MS). Its development was based on previous publications [9-11]. The lower limit of quantitation of the method was 0.0200 ng/mL, and the upper limit of quantitation was 50.0000 ng/mL. Rivastigmine-d_6_ was used as an internal standard.

Pharmacokinetic parameters were derived by means of non-compartmental analysis and were listed and evaluated descriptively by treatment. Primary PK parameters were established under steady-state conditions. They were: AUC_96-264_ (partial area under the plasma concentration *vs.* time for the interval 96h-264h, *i.e.* the time interval of the second and third patch of Test and the fifth to eleventh patch of Reference), Cmax_96-264_ (maximum plasma concentration during the nominal time interval 96h-264h) and Cmin_96-264_ (minimum plasma concentration within the nominal time interval 96h-264h). Other secondary parameters were also calculated (available in Supplementary Material **1**).

Statistical analysis of PK parameters was performed by an analysis of variance (one-way ANOVA; fixed effects of sequence, treatment, period and sequence*subject) and used as the basis for the calculation of 90% confidence intervals, point estimates and confidence intervals for AUC_96-264_, Cmax_96-264_ and Cmin_96-264_ values and the comparison of Test *vs.* Reference were calculated by parametric analysis of the logarithmically transformed values. The three primary PK parameters, AUC_96-264_, Cmax_96-264_ and Cmin_96-264_ were considered as primary decision criteria for bioequivalence assessment. For parametric 90% confidence intervals, acceptance limits of 80%-125% were applied. The point estimate and 90% confidence interval for CTau_264, representing the concentration at the end of the dosing interval of interest 96 - 264 h p.a., were also calculated in a post hoc analysis.

The pharmacokinetic evaluation was carried out by SocraMetrics GmbH. All kinetic parameters were determined model-independently for each treatment using Phoenix^®^ WinNonlin^®^ version 6.3, Phoenix^®^ Connect™ version 1.3.1P software program. The statistical analyses were generated using SAS^®^ (SAS Institute, Cary, NC, USA) version 9.2/9.4 (Mixed procedure).

### 2.4 Sample Size

Based on the results of previous multiple dose studies with similar Test products, the mean intra-subject coefficient of variation of rivastigmine appeared to be CV ≈ 32%. The point estimator for the Test to Reference ratio was about 97% for AUCτ and 102% for Cmax and 93% for Cτ, respectively. Statistically, given that the expected Test to Reference ratio of geometric LS means fell between 94% to 106.4%, it was estimated that the lowest number of subjects to meet the 80.00% to 125.00% bioequivalence range with a statistical power of at least 80% is about N = 48.

For sample size calculation, the hypothesis of adequate adhesion had also been considered. The initial test, according to the EMA guideline (EMA/CPMP/EWP/280/96 Corr1) was the comparison of the mean adhesion *versus* 90%. From data obtained in a previous study, with a Test product similar to the one provided in this trial, the standard deviation of the Test form for adhesiveness was 0.26%. At the lower acceptance limit of 90% the power was about 80% if the mean is greater than 90.095%. The point estimator for the Test product was 99.92% in the previous study C_30410_P1_04 [9], so 48 subjects were considered sufficient, granted that the Test product in the planned trial showed comparable results.

Therefore, considering all data presented above, the inclusion of 58 subjects was sufficient to account for the possibility of a high rate of drop-outs due to adverse events, and variations around the estimated intra-subject CV and to conclude in favor of the hypothesis of bioequivalence with sufficient statistical power.

The treatment sequences for each subject were determined by a randomization list prepared by SocraMetrics GmbH with the help of Minitab^®^ Release 14 software program. The appropriate number of the randomization schedule and associated treatment was allocated for each subject whose entry in the randomized part of the clinical trial was confirmed.

### 2.5 Patch Adhesion

The evaluation of patch adhesion was performed by trained observers after the application of each investigational patch of each treatment. For each of the three Test patches, the assessments were done at 5 min, 12 h, 24 h, 36 h, 48 h, 60 h, 72 h and 84 h and within 5 minutes before removal of the patch (96 h). In the case of the second Test patch, the evaluations ended a day earlier, *i.e.* at 72 hours. For Reference, patch adhesion was assessed at 5 min, 12 h and within 5 minutes before removal of each patch (24 h). According to the EMA Guideline on the pharmacokinetic and clinical evaluation of modified release dosage forms [12] the adhesion was measured as the percentage of area that remained adhered: 0 = ≥ 90% adhered; 1 = ≥ 80% adhered; 2 = ≥ 70% adhered; 3 = ≥ 60% adhered; 4 = ≥ 50% adhered and 5 = <50% adhered or patch completely off the skin.

A statistical analysis of test adhesion was performed at the end of each application, *i.e.* 96 h (end of 1^st^ patch application, 4 days), 168 h (end of 2^nd^ patch application, 7 days) and 264 h (end of 3^rd^ patch application, 11 days). The lower two-sided 90% confidence limit for the mean of Test adhesion was computed by repeated measures analysis with the help of the SAS procedure MIXED. To claim satisfactory adhesion, this confidence limit should be ≥ 90%. Additional-ly, to the patch adhesion (in percentage), the proportion of subjects achieving greater than 90% adherence at each time point and the proportion of subjects with a meaningful degree of detachment were evaluated descriptively.

### 2.6 Safety and Skin Irritation

The safety of treatments was determined primarily by Adverse Event assessment within the study. Body temperature and vital signs (blood pressure and pulse rate) measurements were performed at pre-specified time points. Laboratory parameters and 12-lead ECG were only determined for enrolment and during the end-of-study examination. Safety data were analyzed using descriptive statistics.

In addition, local tolerability and skin irritation were evaluated in accordance with the EMA recommendations [12]. After the removal of each Test patch, the application site was assessed at 30 minutes, 12 hours, 24 hours and 48 hours after each investigational patch removal. For Reference patches, the assessments were performed at 30 min, 12 hours and 24 hours after the removal of each patch and a final assessment was performed 48 hours after removal of the last Reference patch. The evaluation was performed by trained observers according to the scoring system detailed in the EMA Guideline on the pharmacokinetic and clinical evaluation of modified release dosage form [11]. The scoring system measures the dermal response and other effects as follows: *I. Dermal response* as 0. = no evidence of irritation; 1. = minimal erythema, barely perceptible; 2. = definite erythema, readily visible; minimal edema or minimal papular response; 3. = erythema and papules; 4. = definite edema; 5. = erythema, edema, and papules; 6. = vesicular eruption; 7. = strong reaction spreading beyond test site; *II. Other effects* as 0. = none observed; 1. = slightly glazed appearance; 2. = marked glazing; 3. = glazing with peeling and cracking; 4. = glazing with fissures film of dried serous exudates covering all or part of the patch site small petechial erosions and/or scabs. All irritation assessments with a dermal response score of 3 to 7 or any dermal score combined with other effects rating of 4 or greater were reported as Adverse Events.

### 2.7 Pharmacodynamics Sample Collection and Analysis

Blood sampling for assessment of the inhibitory effect of rivastigmine on plasma BuChE activity was performed at -1.5 h and -0.5 h prior to the application of the 1st patch (pre-dose samples) as well as 96 h, 120 h, 144 h, 168 h, 192 h, 216 h, 240 h, 264 h, 266 h, 269 h, 272 h, 288 h after application of the 1st patch. Blood samples were collected in 2mL tubes from a vein using an indwelling cannula with a switch valve or by direct venipuncture. BuChE activity was analyzed by use of a validated fluorescent activity assay using a commercially available kit which is based on a non-fluorescent molecule, ThioStar^®^, that covalently binds to the thiol product of the reaction between the BChE substrate and the BChE in the standards or samples, yielding a fluorescent product read at 510 nm in a fluorescent multimode plate reader with an excitation at 390 nm [13]. Validation followed EMA and FDA standards and covered selectivity, specificity, hook effect and dilutional linearity, linearity, precision, accuracy and stability investigations [14, 15]. The limit of quantitation of the method was 0.313 mU/mL. The inhibitory effect was expressed as the% inhibition of BuChE activity in plasma in comparison to baseline (*i.e.* delta to the pre-dose value in%) and was evaluated descriptively.

## 3 RESULTS

Fifty-eight (58) healthy male subjects were randomized. In each of the two study periods, either 3 patches of Test or 11 patches of Reference with a nominal release rate of rivastigmine of 9.5 mg/24 hours were consecutively applied and worn. The Test patches were worn for 4 days (1st patch), 3 days (2nd patch) and 4 days (3rd patch) and the Reference patches were worn for 24 hours each, thus covering a treatment duration of 11 consecutive days per period.

Due to organizational reasons, subjects were divided in 2 groups. The clinical trial was performed with a cross-over design with intra-individual comparison, thus reducing the variability of the pharmacokinetic parameters. Blood samples for the characterization of pharmacokinetic parameters were collected at predefined time points. Pharmacokinetic parameters for evaluation of bioavailability and assessment of bioequivalence of Test and Reference were calculated at steady-state, *i.e.*, within the application interval of the 2nd and 3rd patch of Test, and the application interval of the 5th to 11th patch of Reference (corresponding to the time interval of 96-264 h post administration). Furthermore, adequate adhesion properties of the Test patch formulation were assessed.

**Fig. (2):**
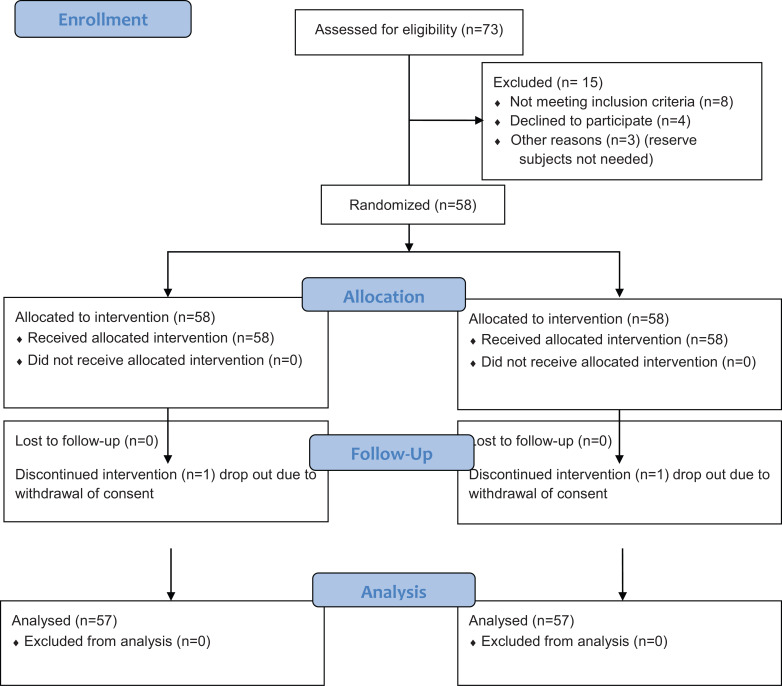
CONSORT diagram.

Of 73 subjects screened, 58 of them were randomly assigned to treatment sequences and all received the study medication (Fig. **2**) of this study which took place between August 22, 2018 (the first subject enrolled) and November 28, 2018 (the last subject completed). As mentioned, male subjects were included, all were Caucasian with a mean age of 41.7 years (FAS). Other demographic data are shown in Table **2**. One (1) subject dropped out from the clinical trial in period II due to withdrawal of consent. The distribution of the subjects in the different analysis populations is shown in Table **3**.

Fifty-seven (57) out of 58 subjects were included in the pharmacokinetic evaluation. Mean pharmacokinetic parameters calculated for rivastigmine are summarized in Table **4** for Test and in Table **5** for Reference. The mean plasma concentration-time profiles (of all homologous values from each cohort) are shown in Fig. (**3**).

Mean curves of rivastigmine plasma concentrations, both after application of the Test product RID-TDS 9.5 mg/24 h (Luye Pharma AG, Germany) and the Reference product Exelon^®^ 9.5 mg/24 (Novartis Pharma GmbH, Germany) indicate a steep increase of concentrations after each patch application. Maximum concentrations are reached within approximately 24 h after each Test patch application and within approximately 9 h after each Reference application. As deduced from the individual profiles, maximum plasma concentrations between approximately 5 ng/mL and 15 ng/mL were measured. In the case of Reference, maximum plasma concentrations were between 4 and 20 ng/mL, with the majority of subjects showing maximum concentrations of about 7-9 ng/mL. A visual comparison of the trough concentrations at the end of the dosing interval of the 1st and the 3rd Test patch (96 h and 264 h post-administration, respectively) indicates very similar values of approximately 4 ng/mL. In contrast and as expected, the concentration at the end of the second dosing interval of the Test is slightly higher (between 5 and 6 ng/mL), reflecting the 24 h shorter application time interval of the 2nd Test patch. Also, in the case of Reference, measured concentrations at the end of each dosing interval of the 11 applied patches were approximately 4 ng/mL.

With the blood sampling scheme in the study (first sample 2 h post administration) no lag-time was observed. After the removal of the last patch of Test and Reference, both mean curves show a rapid decrease in concentrations within the subsequent 12 hours. At the last measurement time point 24 h after removal of the last patch, concentrations were close to or below the LLOQ.

Table **6** shows the parametric point estimates and 90% confidence intervals for the Test *vs.* Reference ratio of the primary pharmacokinetic parameters. Calculated point estimates and 90% confidence intervals for all primary target PK parameters are within the predefined acceptance interval of 80.00-125.00% for bioequivalence assessment.

**Fig. (4):**
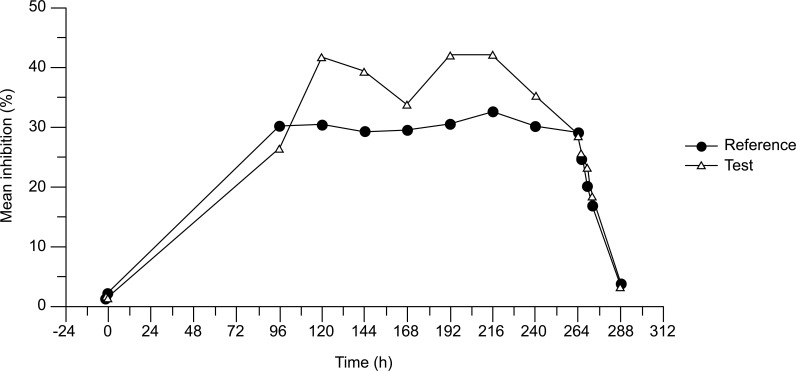
Inhibition of butyryl cholinesterase (BuChE) plasma activity over time (of all homologous values from the cohorts

Mean inhibition of BuChE activity in plasma during the observation period of primary interest (96-264 h post administration) was nearly identical for Test and Reference at the common trough time points 96, 168 and 264 h post-administration, remaining around 30% relative to baseline (Fig. **4**). Mean curve of BuChE activity inhibition for Test reached at a slightly higher level of about 40% only at those sampling points, where Reference was at the end of a dosing interval (trough points) and Test was still within a treatment interval. Trough BuChE inhibition for the Test did not fall below that of Reference, indicating that the prolonged dosing interval did not result in any type of adaption. Within the steady state phase, for Test a similar ratio between the maximum and minimum BuChE inhibition was observed as for the Reference product.

All 58 randomized subjects were included in the evaluation of patch adhesion. As shown in Table **7**, inferential statistical analysis yielded a point estimate of 96.90% for the mean adhesion of the Test patches at the end of the dosing interval with a corresponding 90% confidence interval of 94.71-99.09%. Based on these results, as the lower limit of the confidence interval lies above 90%, satisfactory adhesion has been demonstrated for the Test. For Reference, this criterion is not met, that is Reference shows a slightly higher degree of patch detachment when compared to Test.

**Fig. (5):**
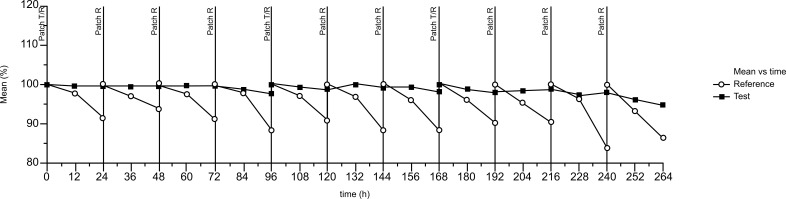
Mean adhesion percentage of patches.

The descriptive evaluation indicated a mean percentage of adhesion of 98.74% for Test and 95.29% for Reference. Mean curves showed a nearly 100%-adhesion during the application time of Test patches and a decline of mean adhesion during the application of the daily Reference patches (Fig. **5**). At the time point, 24 h post-administration Test patches showed adherence of 90% or greater in 94.83% of the assessments, whereas for Reference patches ≥ 90% adherence was assigned for 66.77% of the total observations. At the subsequent evaluation, time points 36 h, 48 h, 60 h, 72 h, 84 h, and 96 h. post administration, relevant for the Test patches only, an adherence of ≥ 90% was assigned for 88-97% of the observations.

There were no cases of complete patch detachments for none of the treatments. Furthermore, during Test treatment score 5 (< 50% adhered or patch completely off the skin) was assigned for none of the patches, while for Reference score 5 was assigned in 1.36% of the cases (26 assessments in several subjects).

A total of 58 subjects received at least one IMP and were included in the evaluation of safety. One (1) subject dropped out in the 2nd period (study day 9) during the treatment with Test patches. For the remaining 57 subjects drug exposure was in accordance with the protocol. Local tolerability results are reported in Table **8**. No scores ranging from 4 to 7 were observed.

Over the complete assessment period, mean dermal response at the individual assessment time points ranged from 0.7 to 1.9 for Test and from 0.6 to 1.9 for Reference. Results are shown in Fig. (**6**), where no difference is observed between the dermal response assessed after the removal of the first patch and the dermal response after the removal of the last patch for both treatments. Furthermore, the mean curve shows that skin reactions observed immediately after patch removal improved over time.

With regard to the local tolerability ratings of “other effects” after patch removal, in the vast majority of cases, score 0 (“none observed”) was assigned for Test and Reference. In the case of Test, for 1 subject scoring 4 was assigned for the finding “application site erosion” observed shortly after the removal of the last patch. This unexpected finding was only mild and resolved in the evening of the same day.

Forty-three (43) out of 58 (74.14%) subjects reported at least one AE during the study. In total 149 AEs were reported and 97 (65.10%) were assessed as related. Out of the 97 IMP-related AEs reported throughout the study, 40 AEs (41.24%) were assessed as related to the Test product and 57 AEs (58.76%) were assessed as related to the Reference product. The intensity of the IMP-related AEs reported during the application of Test patches was mild in 33 out of 40 cases and moderate in 7 out of 40 IMP-related AEs. In comparison, during the application of Reference patches, out of the 57 IMP-related AEs 42 AEs were of mild intensity, 13 AEs were of moderate intensity and 2 headache AEs were rated as severe intensity. The most frequent IMP-related AEs were “application site erythema” (20 reports in 13 subjects) and “headache” (20 reports in 10 subjects). No serious AEs occurred and no subjects dropped out due to AEs.

In general, no clinically relevant changes in the laboratory values, ECG parameters, vital signs and physical parameters related to IMPs’ safety profile were observed during the study and between screening and end of study examination, as per investigator’s criteria.

## 4 DISCUSSION

This clinical trial was conducted to compare the bioavailability of rivastigmine and assess bioequivalence between the Test product RID-TDS 9.5 mg/24 h (bi-weekly patch) and the marketed Reference product Exelon^®^ 9.5 mg/24 hours transdermal patch (daily patch) under steady-state conditions. The Test product is currently the only multiday patch authorized in the market. Furthermore, adequate adhesion properties of the Test patch formulation were assessed. As secondary objectives, the inhibitory effect of Test and Reference on endogenous plasma BuChE was determined and general safety and local tolerability of the IMPs were evaluated in the trial population. Results of the Test multiday patch, clearly demonstrated activity through BuChE inhibition, bioequivalence compared to daily treatment, good adherence, and good cutaneous and systemic tolerability in bioequivalence studies. Rivastigmine patch twice a week will provide a clinical benefit to a specific profile of patients and in specific situations, facilitating adherence to treatment by reducing the patch application frequency, minimizing medication errors and reducing the caregiver's burden.

Calculated point estimates and 90% confidence intervals for all primary target parameters AUC96-264, Cmax96-264 and Cmin96-264 as well as CTau_264, representing trough concentrations at the end of the dosing interval of interest 96-264 h post administration were within the predefined acceptance interval of 80.00-125.00% for bioequivalence assessment (see Table **6**). Therefore, statistical evaluation of the primary pharmacokinetic parameters demonstrated bioequivalence between the Test product and the marketed Reference transdermal patch under steady-state conditions. Despite the longer dosing interval, absolute minimum concentrations after the Test were comparable to Reference and, if deviating, only slightly higher. Moreover, trough concentrations at the end of the dosing interval of interest 96 – 264 h p.a. were also very similar for both treatments. Accordingly, no risk of therapeutic failure is to be expected.

Satisfactory adhesion for Test has been demonstrated based on the results of statistical analysis of mean adhesion at the end of the dosing interval. In addition, Test showed a better adherence on the skin during the study in comparison to the Reference, based on descriptive analysis. Regarding the assessments and AEs referring to local tolerability, the difference in absolute numbers between Test and Reference is partly owed to the fact that for Test local tolerability assessments were performed 12 times per subject, whereas for Reference local tolerability was assessed much more often (34 times per subject).

Within the steady state phase, mean inhibition of BuChE plasma activity was nearly identical for Test and Reference at the common trough time points 96, 168 and 264 h p.a., remaining around 30% relative to baseline. Trough BuChE inhibition for Test did not fall below that of Reference, indicating that the prolonged dosing interval did not result in any type of adaption. During the observation period of primary interest, the mean inhibition of plasma BuChE activity over time was nearly identical for both products. Furthermore, regarding the BuChE inhibition under steady-state conditions, inhibition at the beginning and at the end of the Total observation period was practically the same for both treatments. This consistent and quantitative evidence is a good indicator of the similar clinical efficacy of both administrations. A pharmacokinetic/pharmacodynamic modeling of rivastigmine transdermal patch was already performed for the Reference patch and considering that similar results have been observed with the Test transdermal patch, no further studies were conducted [16].

Both products were transdermal patches of matrix-type, where the drug is directly entrapped in a polymer matrix. The Test product is composed of multiple layers. This drug-containing patch contains a backing film to protect the patch from the environment, a polymeric matrix loaded with rivastigmine to allow release for up to 4 days, a polymeric film that governs the drug release, a skin-facing adhesive layer, and a release liner that is removed prior to application. As mentioned, both products offered a nominal release rate of 9.5 mg rivastigmine per 24 h, with the added value that the newly developed Test product was a twice weekly patch (application duration of 4 or 3 days), while the Reference product was a once-daily patch.

For the present bioavailability and bioequivalence study, a cross-over design was selected following recommendations by the Committee for Medicinal Products for Human Use (CHMP) [17]. Proper randomization of subjects to the sequence of formulation administration provides the best-unbiased estimates for the differences (or ratios) between formulations. According to data from the literature the elimination half-life of rivastigmine as a transdermal patch formulation has been determined as approximately 3 hours [18, 19]. Furthermore, in a recently conducted clinical trial, the mean apparent elimination half-life in plasma rivastigmine for Reference was determined as 10.93 h (3.77 h-15.40 h) and for Test as 8.13 h (2.33 h-12.08 h) [20, 21]. Thus, a washout period of 14 days between the last patch removal of the first treatment and the first patch application of the subsequent treatment was sufficient for completely eliminating the active ingredient from the previous treatment, excluding any carryover effects between periods.

For the bioequivalence assessment 57 subjects who completed the study according to the protocol were considered. Mean plasma concentration *vs.* time curves of rivastigmine after application of the Test product and the Reference product both show an initial relatively steep increase after each patch application. Maximum concentrations are reached within approximately 24 h after each Test patch application and within approximately 9 h after each Reference application. Mean and individual curves well reflect the diverging dosing intervals, twice weekly for Test and once daily for Reference. For the decision upon bioequivalence the 7-day-treatment period under steady-state conditions was used by comparing AUC_96-264_, Cmax_96-264_, and Cmin_96-264_. According to available data steady state is reached after first application of the 4-day Test patch as well as after 4 days of Reference applications [9]. Thus, for equivalence assessment, the second and the third Test patches were compared with the Reference patches from 5th to 11th.

It must be considered that Alzheimer's disease, like the rest of dementias, generates a significant burden on the family members or caregiver. The social, economic and family situations vary greatly in each case between the patient/caregiver/family, so the treatment must be as personalized as possible. The rivastigmine patch twice a week will be another alternative that the specialist will be able to offer to the patient, the caregiver and the patient's family in the treatment of Alzheimer's disease. For the convenience of use, it is targeted that the Test patch is replaced alternating after 4 days / 3 days, enabling two fixed days of patch change a week, *i.e.* each Monday and Friday morning. The twice-a-week patch requires a less frequent application with a better guarantee of receiving rivastigmine in a constant way and facilitating the treatment compliance of the patient. Additionally, the Test patch requires less planning and logistics of the patients and caregivers (mainly during the weekend) and thus less burden on the caregiver. Patient and caregiver satisfaction is essential for good adherence to treatment.

The clinical trial has strengths and limitations. The strength is the large population of subjects evaluated, larger than the calculated following statistical criteria defining sample size. One of the limitations of the study is that it has been performed in healthy subjects and that the Test patch has to be evaluated clinically to confirm some of the alleged benefits.

## CONCLUSION

In summary, the pharmacokinetic evaluation demonstrated bioequivalence between the Test patch formulation RIV-TDS 9.5mg/24h and the marketed Reference product Exelon^®^ 9.5mg/24hours transdermal patch under steady-state conditions *i.e.* for Test with 2 patches (3 days and 4 days) and Reference with 7 daily patches. Despite the longer dosing interval absolute minimum concentrations after Test were comparable to Reference and, if deviating, only slightly higher. Moreover, trough concentrations at the end of the dosing interval of interest 96 – 264 h p.a. were also very similar for both treatments. The variability observed in pharmacokinetic parameters for the two patches tested was very similar. Accordingly, no risk of therapeutic failure is to be expected. Furthermore, total and maximum exposure were comparable, so that in the end highly similar therapeutic efficacy, as well as adequate safety, is to be deduced from these data.

A satisfactory patch adhesion for Test, was confirmed to be fully in line with the requirements as laid down in EMA [11]. Remarkably, Test shows better adhesion properties despite the longer dosing interval and - of importance - the better adhesion properties do not come along with impaired local tolerability. Thus, as a consequence of these combined observations, adhesion and tolerability properties of Test support the favorable *in vivo* performance of the new rivastigmine multiday patch.

Systemic tolerability of Test and Reference was in accordance with the safety profile of the drug substance. The majority of reported AEs were of mild intensity. No serious AEs occurred and no subjects dropped out due to AEs. With regard to local tolerability assessment after patch removal, both treatments were well tolerated and the observed skin reactions improved over time. For Test, one local finding (“application site erosion”) was assessed as unexpected, however, it was only mild and rapidly resolved. Both bioequivalence and pharmacodynamic evidence of BuChE inhibition support fully the clinical and therapeutic efficacy of the rivastigmine patch twice a week.

## Figures and Tables

**Fig. (3):**
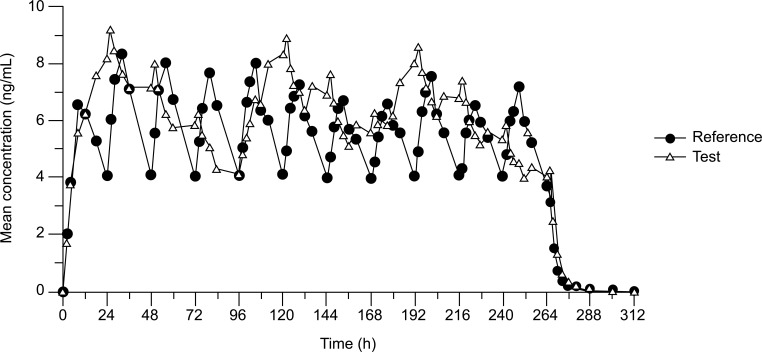
Rivastigmine mean plasma concentrations over time (of all homologous values from the cohorts).

**Fig. (6):**
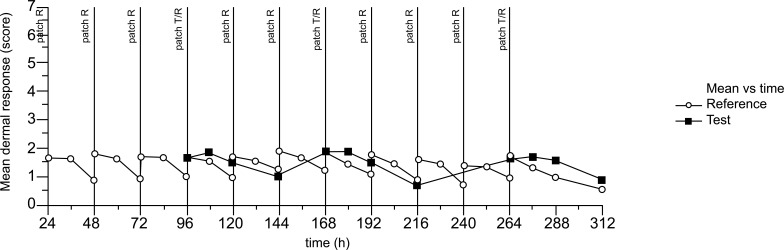
Skin irritation induced by transdermal patches.

**Table 1:** PK sample collection and statistical analysis.

-	**Day 1**	**Day 2**	**Day 3**	**Day 4**	**Day 5**	**Day 6**	**Day 7**	**Day 8**	**Day 9**	**Day 10**	**Day 11**
**Test**	**4 Day Test Patch**				**3 Day Test Patch**			**4 Day Test Patch**			
**Reference**	Reference patch	Reference patch	Reference patch	Reference patch	Reference patch	Reference patch	Reference patch	Reference patch	Reference patch	Reference patch	Reference patch
**PK Determinations (Hours from the First Patch Application)**	0, 2, 4, 8, 12, 18 h	24, 26, 28, 32, 36 h	48, 50, 52, 56, 60 h	72, 74, 76, 80, 84 h	96, 98, 100, 102, 105, 108, 112 h	120, 122, 124, 126, 129, 132, 136 h	144, 146, 148, 150, 153, 156, 160 h	168, 170, 172, 174, 177, 180, 184 h	192, 194, 196, 198, 201, 204, 208 h	216, 218, 220, 222, 225, 228, 232 h	240, 242, 244, 246, 249, 252, 256, 264, 266, 268, 270, 273, 276, 280, 288, 300, 312 h

**Table 2:** Demographic data-statistics, per analysis set.

**Analysis st: FAS = SafAS = PPS-PA**				
**Parameter**	**Age (years)**	**Height (m)**	**Weight (kg)**	**BMI (kg/m2)**
N	58	58	58	58
Arithmetic Mean	41,7	1,793	82,61	25,71
Standard Deviation	8,2	0,071	8,71	2,33
Minimum	28	1,62	65,5	20,40
Median	42,0	1,790	82,00	25,90
Maximum	55	1,99	104,0	29,80
**Analysis st: PPS-PK**				
**Parameter**	**Age (years)**	**Height (m)**	**Weight (kg)**	**BMI (kg/m2)**
N	57	57	57	57
Arithmetic Mean	41,9	1,793	82,58	25,7
Standard Deviation	8,2	0,071	8,78	2,34
Minimum	28	1,62	65,5	20,4
Median	42,0	1,790	82	25,8
Maximum	55	1,99	104,0	29,8

**Table 3:** Data sets analyzed.

**Data Set**	**Number of Subjects Included**	**Number of Subjects Excluded**	**Random Number of Subjects Excluded**
FAS	58	-	-
SafAS	58	-	-
PPS-PA	58	-	-
PPS-PK	57	1	1

**Table 4:** Mean pharmacokinetic parameters of rivastigmine after multiple applications of 3 consecutive patches of RID-TDS 9.5 mg/24 h (Test) for a total of11 days (4-3-4) to 57 subjects (104.5 mg rivastigmine per treatment).

**-**	AUC 96-264 (h*ng/mL)	**Cmax 96-264 (ng/mL)**	**Cmin 96-264 (ng/mL)**	AUC 96-168 (h*ng/mL)	AUC 168-264 (h*ng/mL)	AUC apr (h*ng/mL)
N	57	57	57	57	57	57
Mean	1060	9,92	3,03	481	576	26,50
SD	340	2,99	1,13	164	181	9,63
Min	360	4,70	0,602	135	224	8,26
Median	1000	9,30	3,00	457	538	24,50
Max	2040	18,4	6,02	959	1080	52,80
Geometric mean	1000	9,49	2,81	453	549	24,80
CV% G. Mean	34,14	31,17	43,58	37,52	32,7	38,61

**Table:** 

**-**	tmax 96-264 (h*ng/mL)	**tmin 96-264 (ng/mL)**	**Clast (ng/mL)**	Ctau_96 (h*ng/mL)	Ctau_168 (h*ng/mL)	Ctau_264 (h*ng/mL)	**tlast (h)**	**t1/2 (h)**	**Lz (1/h)**
N	57	57	57	57	56	57,00	57	57	57
Mean	143	179	0,0386	4,13	5,59	4,06	300	8,06	0,09645
SD	36,3	69,2	0,0176	1,40	1,91	1,31	9,62	2,73	0,03392
Min	100	96,0	0,0207	1,79	2,34	1,37	288,00	3,49	0,04826
Median	122	174,00	0,0344	3,93	5,19	3,91	300,00	7,72	0,08983
Max	232	264	0,1010	8,79	11,40	7,76	312,00	14,4	0,19884
Geometric mean	139	165	0,0356	3,91	5,27	3,86	300,00	7,62	0,09101
CV% G. Mean	24,10	43,96	40,8	35,13	36,66	32,95	3,21	35,32	35,32

**Note:** AUC96-264: partial area under the plasma concentration *vs.* time profile for the time interval 96 h-264 h, *i.e.* the time interval of the second and third patch of Test/the fifth to eleventh patch of Reference (aka days 5 – 11), calculated by means of the linear trapezoidal method (linear trapezoidal rule).

Cmax,96-264: maximum concentration in plasma during the nominal time interval 96 h-264 h, obtained directly from measured values

Cmin,96-264: (absolute) minimum concentration within the nominal time interval 96 h-264 h, obtained directly from measured values

AUC96-168: partial area under the plasma concentration *vs.* time profile for the time interval 96 h-168 h, *i.e.* the time interval of the second patch of Test/the fifth to seventh patch of Reference (aka days 5 – 7), calculated by means of the linear trapezoidal method (linear trapezoidal rule)

AUC168-264: partial area under the plasma concentration *vs.* time profile for the time interval 168 h-264 h, *i.e.* the time interval of the third patch of Test/the eight to eleventh patch of Reference (aka days 8 – 11), calculated by means of the linear trapezoidal method (linear trapezoidal rule)

AUC apr: partial area after removal of the last patch calculated as area under the curve starting at the sample time at the nominal time point 264 h until tlast

tmax,96-264: Time to reach the maximum concentration during the nominal time interval 96 h-264 h

tmin,96-264: Time to reach the minimum concentration during the nominal time interval 96 h-264 h

Clast: concentration at the last time point with quantifiable concentration (C ≥ LLOQ)

Ctau_96: trough concentration at the planned time point 96 h p.a., obtained directly from measured values

Ctau_168: trough concentration at the planned time point 168 h p.a., obtained directly from measured values

Ctau_264: trough concentration at the planned time point 264 h p.a., obtained directly from measured values

Tlast: last time point with quantifiable concentration (after last patch removal)

t1/2: apparent terminal half-life, t1/2 = ln(2) / λ (after last patch removal)

Lz: apparent terminal rate constant determined by log-linear regression (after last patch removal)

**Table 5:** Mean pharmacokinetic parameters of rivastigmine after multiple applications of 11 consecutive daily patches of Exelon^®^ 9.5 mg/24 h (Reference) for a total of 11 days (4-3-4) to 57 subjects (104.5 mg rivastigmine per treatment).

**-**	AUC 96-264 (h*ng/mL)	**Cmax 96-264 (ng/mL)**	**Cmin 96-264 (ng/mL)**	AUC 96-168 (h*ng/mL)	AUC 168-264 (h*ng/mL)	AUC apr (h*ng/mL)
N	57	57	57	57	57	57
Mean	937	9,75	2,87	411	526	18,80
SD	330	3,92	1,15	155	181	5,64
Min	417	3,78	0,429	178	236	8,21
Median	911	9,34	2,74	405	521	18,60
Max	1990	22,9	5,46	889	1100	31,70
Geometric mean	884	9,03	2,61	385	497	18,00
CV% G. Mean	35,68	41,01	49,55	38,02	34,83	32,13

**Table:** 

**-**	tmax 96-264 (h*ng/mL)	**tmin 96-264 (ng/mL)**	**Clast (ng/mL)**	Ctau_96 (h*ng/mL)	Ctau_168 (h*ng/mL)	Ctau_264 (h*ng/mL)	**tlast (h)**	**t1/2 (h)**	**Lz (1/h)**
N	57	-	-	-	-	-	-	-	-
Mean	171	190	0,0369	4,07	3,99	3,73	3,05	12,7	0,06388
SD	55,5	49,2	0,0150	1,37	1,30	1,11	9,35	4,89	0,02639
Min	100	96,0	0,0201	0,0997	1,82	1,23	288	4,94	0,02920
Median	184	196	0,0329	3,90	3,67	3,51	312	11,9	0,05818
Max	252	264	0,1140	8,76	8,33	6,89	312	23,7	0,14041
Geometric mean	162	183	0,0347	3,79	3,79	3,56	305	11,7	0,05903
CV% G. Mean	34,91	29,85	34,36	32,64	32,64	32,65	3,11	41,48	41,48

**Table 6:** Parametric point estimates and 90% confidence intervals determined for the primary pharmacokinetic parameters of rivastigmine; comparison Test *vs.* Reference.

**Parameter**	**Point Estimate (%)**	**90% Confidence Interval (%)**	**CV%**
AUC 96-264	113,64	107,33-120,33	18,39
Cmax, 96-264	105,14	98,38-112,38	21,46
Cmin, 96-264	107,82	97,78-118,89	31,96
Ctau_264	108,60	101,81-115,64	20,82

**Table 7:** Point estimates and 90% confidence intervals determined for mean adhesion at the end of application interval of Test and Reference, N=811 observations.

**Least Squares Means**								
**Treatment**	**Estimate**	**Standard Error**	**DF**	**t Value**	**Pr > t**	**Alpha**	**Lower**	**Upper**
Reference	89,3774	1,0299	62,2	86,78	<0.001	0,1	87,6576	91,0971
Test	96,8988	1,3231	163	73,24	<0.001	0,1	94,7100	99,0875

**Table 8:** Local tolerability results.

**Score**	**Local Tolerability**	**Test**	**Refs.**
0	No evidence of irritation	8,19% (57)	10,4% (205)
1	Minimal erythema, barely perceptible	36,78% (256)	43,05% (849)
2	Definite erythema, readily visible; minimal edema or minimal papular response	53,16% (370)	45,13% (890)
3	Erythema and papules	1,01% (7)	1,42% (28)

## Data Availability

Not applicable.

## LIST OF ABBREVIATIONS

AD: Alzheimer's Disease
NFTs: Neurofibrillary Tangles
AChEIs: Acetylcholinesterase Inhibitors
