# Supplementary material for: Comparative Bioavailability Study of a Novel Multi-Day Patch Formulation of Rivastigmine (Twice Weekly) with Exelon® Transdermal Patch (Daily)- A Randomized Clinical Trial
Source: Curr Alzheimer Res. 2022 Nov 10;19(7):541–53. doi: 10.2174/1567205019666220823105059 (PMC10186381; doi:10.2174/1567205019666220823105059)
Supplement: Supplementary file 1 [file CAR-19-541_SD1.pdf]

# Supplementary Materials

# Comparative Bioavailability Study of a Novel Multi-Day Patch Formulation of Rivastigmine (Twice Weekly) with Exelon® Transdermal Patch (Daily)- A Randomized Clinical Trial

Bjoern Schurad[1], Cornelius Koch[1,2], Barbara Schug[2], Adelaida Morte[3], Anna Vaqué[3], Rafael De la Torre[4] and Marc Iniesta[3,*]

*[1]Luye Pharma AG, Miesbach, Germany; [2]SocraTec R&D GmbH, Erfurt, Germany; [3]ESTEVE Pharmaceuticals SA, Barcelona, Spain; [4]Research Group in Integrated Pharmacology and Systems Neuroscience, Hospital del Mar Research Institute Doctors (IMIM), Barcelona, Spain*

## SUPPLEMENTARY MATERIAL 1

### Secondary pharmacokinetic parameters

The following secondary PK parameters were also calculated: $AUC_{96\text{-}168}$ (partial area under the plasma concentration *vs.* time for the interval 96h-168h, *i.e.* the time interval of the second patch of Test or the fifth to seventh patch of Reference), $AUC_{168\text{-}264}$ (partial area under the plasma concentration *vs.* time for the interval 168 h-264 h, *i.e.* the time interval of the third patch of Test or the eight to eleventh patch of Reference), AUCapr (partial area after removal of the last patch), $tmax_{96\text{-}264}$ (Time to reach the maximum concentration during the interval 96h-264h), $tmin_{96\text{-}264}$ (Time to reach the minimum concentration during the interval 96h-264h), $C_{last}$ (concentration at the last time point with quantifiable concentration ($C \geq LLOQ$), $C\tau_{\_96}$ (trough concentration at the planned time point 96 h), $C\tau_{\_168}$ (trough concentration at the planned time point 168 h), $C\tau_{\_264}$ (trough concentration at the planned time point 264 h), tlast (last time point with quantifiable concentration, after last patch removal), t1/2 (apparent terminal half-life) and $\lambda Z$ (apparent terminal rate constant).

1875-5828/22 © 2022 Bentham Science Publishers
